# A novel chromatin-remodeling complex variant, dcPBAF, is involved in maintaining transcription in differentiated neurons

**DOI:** 10.3389/fcell.2023.1271598

**Published:** 2023-11-14

**Authors:** Nataliya V. Soshnikova, Asya M. Azieva, Nataliya S. Klimenko, Alvina I. Khamidullina, Alexey V. Feoktistov, Andrey A. Sheynov, Alexander V. Brechalov, Victor V. Tatarskiy, Sofia G. Georgieva

**Affiliations:** ^1^ Department of Transcription Factors, Engelhardt Institute of Molecular Biology, Russian Academy of Sciences, Moscow, Russia; ^2^ Center for Precision Genome Editing and Genetic Technologies for Biomedicine, Engelhardt Institute of Molecular Biology, Russian Academy of Sciences, Moscow, Russia; ^3^ Department of Eukaryotic Transcription Factors, Institute of Gene Biology, Russian Academy of Sciences, Moscow, Russia; ^4^ National Research Center “Kurchatov Institute”, Moscow, Russia; ^5^ Center for Precision Genome Editing and Genetic Technologies for Biomedicine, Institute of Gene Biology, Russian Academy of Sciences, Moscow, Russia; ^6^ Department of Molecular Oncobiology, Institute of Gene Biology, Russian Academy of Sciences, Moscow, Russia

**Keywords:** neuronal differentiation, PHF10A, PHF10D, chromatin remodeling, PBAF complex, dcPBAF, PHF10 isoforms, double PHD finger domain

## Abstract

The Polybromo-associated BAF (BRG1- or BRM-associated factors) (PBAF) chromatin-remodeling complex is essential for transcription in mammalian cells. In this study, we describe a novel variant of the PBAF complex from differentiated neuronal cells, called dcPBAF, that differs from the canonical PBAF existing in proliferating neuroblasts. We describe that in differentiated adult neurons, a specific subunit of PBAF, PHF10, is replaced by a PHF10 isoform that lacks N- and C-terminal domains (called PHF10D). In addition, dcPBAF does not contain the canonical BRD7 subunit. dcPBAF binds promoters of the actively transcribed neuron-specific and housekeeping genes in terminally differentiated neurons of adult mice. Furthermore, in differentiated human neuronal cells, PHF10D-containing dcPBAF maintains a high transcriptional level at several neuron-specific genes.

## Introduction

Two evolutionarily conserved subfamilies of the SWI/SNF type chromatin-remodeling complexes, PBAF and BAF, play comprehensive and diverse roles in the transcriptional regulation of metazoans, which are being involved in transcription, development, and differentiation (Wu, 2012). These complexes contain common subunits, namely, the mutually exclusive ATPases (BRG1 or BRM), other common core subunits, and complex-specific subunits: the BAF complex contains BAF250 and DPF1,2,3, whereas the PBAF complex contains BAF200, BAF180, and PHF10/BAF45a (Centore et al., 2020). These two subfamilies also differ in their functions (Hodges et al., 2016).

Metazoan BAF and PBAF complexes were purified by conventional chromatographic methods from proliferating cells, including *Drosophila* embryos and mammalian cell cultures (Xue et al., 2000; Yan et al., 2005; Vorobyeva et al., 2009). In addition, several types of non-canonical BAF complexes, which differ in subunit composition from PBAF and control specific sets of genes, were described in mice. They are the mouse nPBAF, which are essential for the proliferation of stem/progenitor cells of mammalian CNS (Lessard et al., 2007), and the esBAF, which is a murine embryonic stem cell (ESC) chromatin-remodeling complex, which maintains the pluripotent state of ESCs (Ho et al., 2009). Moreover, the small non-canonical GBAF complex regulates naive pluripotency in mouse ESCs (Gatchalian et al., 2018). BAF subunits specific for differentiated cells, in particular neuron-specific BAF subunits, have also been described (Vogel-Ciernia et al., 2013; Sokpor et al., 2017). Nevertheless, all of these canonical and non-canonical complexes were purified from proliferating precursors, whereas SWI/SNF-type complexes from differentiated cells have not yet been purified.

PHF10 is a specific subunit of the mammalian and *Drosophila* PBAF chromatin-remodeling complex (Chalkley et al., 2008; Vorobyeva et al., 2009; Brechalov et al., 2014). The mouse knockout of PHF10 and mutations of the PHF10 homolog-encoding gene in *Drosophila* are embryonic lethal (Shidlovskii et al., 2005; Krasteva et al., 2017). It was recently shown that PHF10 cooperates with MYC in the transcriptional activation of genes responsible for proliferation (Soshnikova et al., 2021).

PHF10 together with two other PBAF-specific subunits, BRD7 and PBRM1, forms a histone N-tail recognition submodule unique to PBAF (Yuan et al., 2022). BRD7 and PBRM1 contain bromodomains (BDs) which bind acetylated lysine residues on histones, whereas PHF10 contains the C-terminal double PHD finger (DPF) domain. The PHD domains of DPF are tandemly organized in a face‐to‐back manner in a single structure that interacts with histone N‐termini differently from a single PHD (Local et al., 2018; Soshnikova et al., 2020; Chugunov et al., 2021).

Previously, we have found that mouse and human PHF10 have four isoforms which arise as a result of transcription from two alternative promoters and the existence of two different transcription termination sites. PHF10 isoforms alternatively incorporate into the PBAF complex (Brechalov et al., 2014). Of them, PHF10A and PHF10D isoforms appear to be the most functionally important, as they have been shown to maintain transcription in mammalian cells of the opposite differentiation status. The DPF-containing isoform PHF10A, also described as BAF45a, is required for the proliferation of mouse neural progenitors (Lessard et al., 2007), the maintenance of adult mouse hematopoietic stem cells (Krasteva et al., 2017), and the proliferation of human myeloid progenitors (Viryasova et al., 2019). The PHF10D isoform lacks DPF and 46 N-terminal amino acids, is the only PHF10 isoform present in differentiated mature human neutrophils, and is responsible for maintaining the transcription of neutrophil-specific genes (Viryasova et al., 2019).

Here, we studied the composition and function of the PBAF complex and its PHF10A and PHF10D subunit variants in postnatal mammalian brain development. We have found that PHF10 is highly expressed in neurons but not in other cells of the adult mouse brain. We further purified the neuron-specific PBAF complex from newborn and adult mice using chromatographic and immunoaffinity approaches. We identified a novel variant of the PBAF complex specific for differentiated neuronal cells (hereafter called dcPBAF), which replaces the canonical PBAF complex in adult mouse neurons. dcPBAF lacks the canonical BRD7 and PHF10A subunits but contains a PHF10D subunit. The expression of PHF10A, which is dominant in the neurons of the postnatal mouse brain, dropped down following the transition from the proliferative to the differentiation stage. At the same stage, the expression of PHF10D increased, and it became the only PHF10 isoform present in the terminally differentiated neurons of 4-month-old adult mice (P120). dcPBAF associates with promoters of actively transcribed housekeeping and neuron-specific genes. Moreover, we found that in human differentiated neurons, the PHF10D isoform is also dominantly expressed. This isoform replaces PHF10A isoforms upon the commencement of differentiation in SH-SY5Y immortalized human neuroblastoma cells and maintains transcription of the genes specific for differentiated neurons.

## Results

### The distinct type of the PBAF complex which contains the PHF10D isoform becomes dominant in differentiated neurons

**FIGURE 1:**
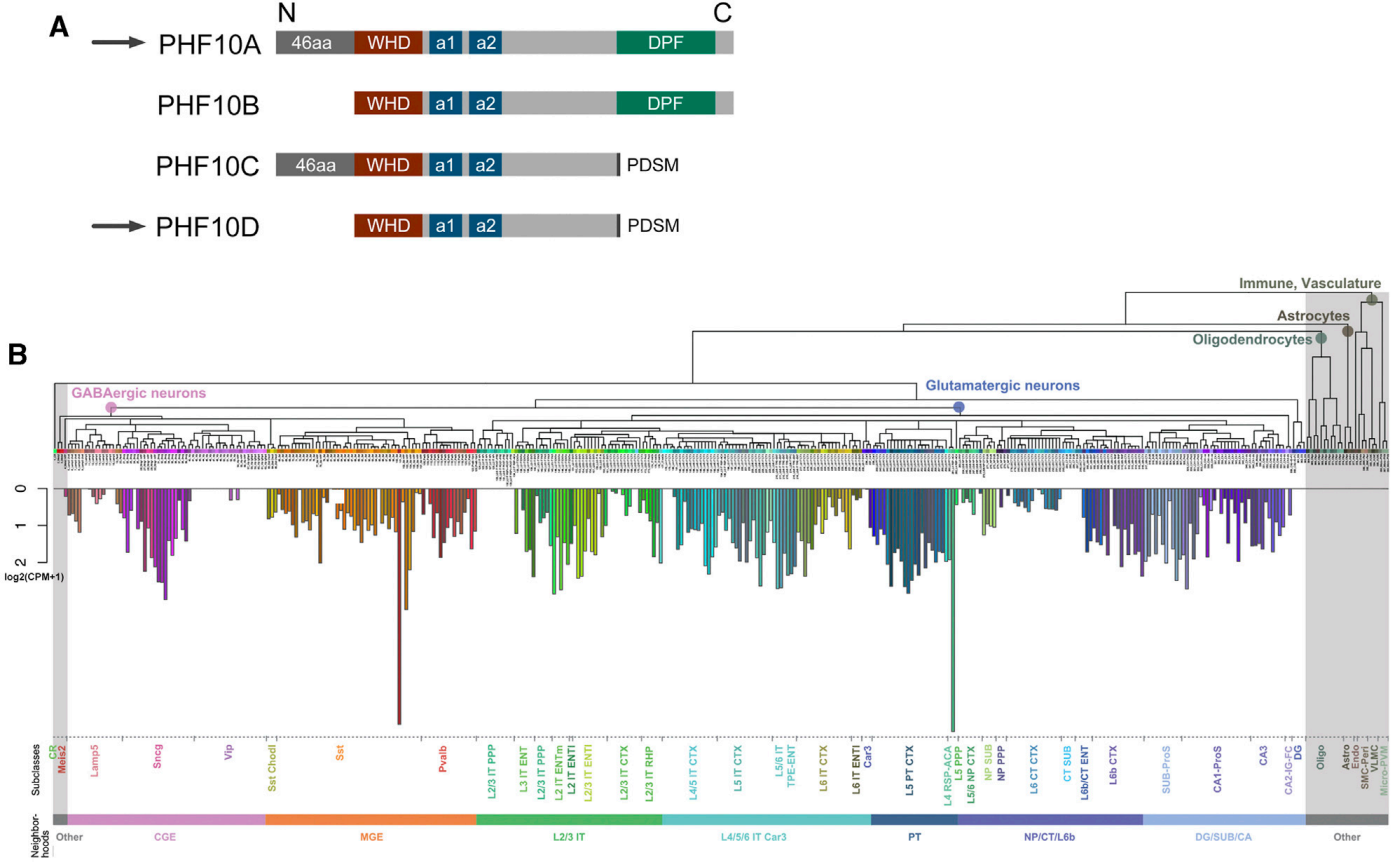
**(A)** Schematic representation of human and mouse PHF10 isoforms. **(A)** Schematic representation of PHF10 protein isoforms. All isoforms contain WHD (dark red box), alpha-1 (a1), and alpha-2 (a2) (dark blue boxes) structured domains. The PHF10A isoform contains a C-terminal DPF (dark green boxes) and 46 N-terminal amino acids. PHF10B is identical to PHF10A but lacks the N-terminal amino acids. PHF10C and PHF10D isoforms also differ from each other in the presence of N-terminal amino acids. Black rectangles indicate the C-terminal phosphorylation-dependent sumoylation motif (PDSM) which is present in PHF10C and PHF10D. PHF10A and PHF10D isoforms that are the subject of this study are indicated by arrows. In our previous studies, PHF10A, B, C, and D isoforms were designated as PHF10-Pl, PHF10-Ps, PHF10-Sl, and PHF10-Ss, respectively. **(B)** Mean expression of PHF10 in different cell types of isocortex and hippocampal formation. Non-neuronal cells are highlighted in gray. A more detailed description of different brain parts, cell types, and characterization can be found at https://doi.org/10.1016/j.cell.2021.04.021.

**FIGURE 2:**
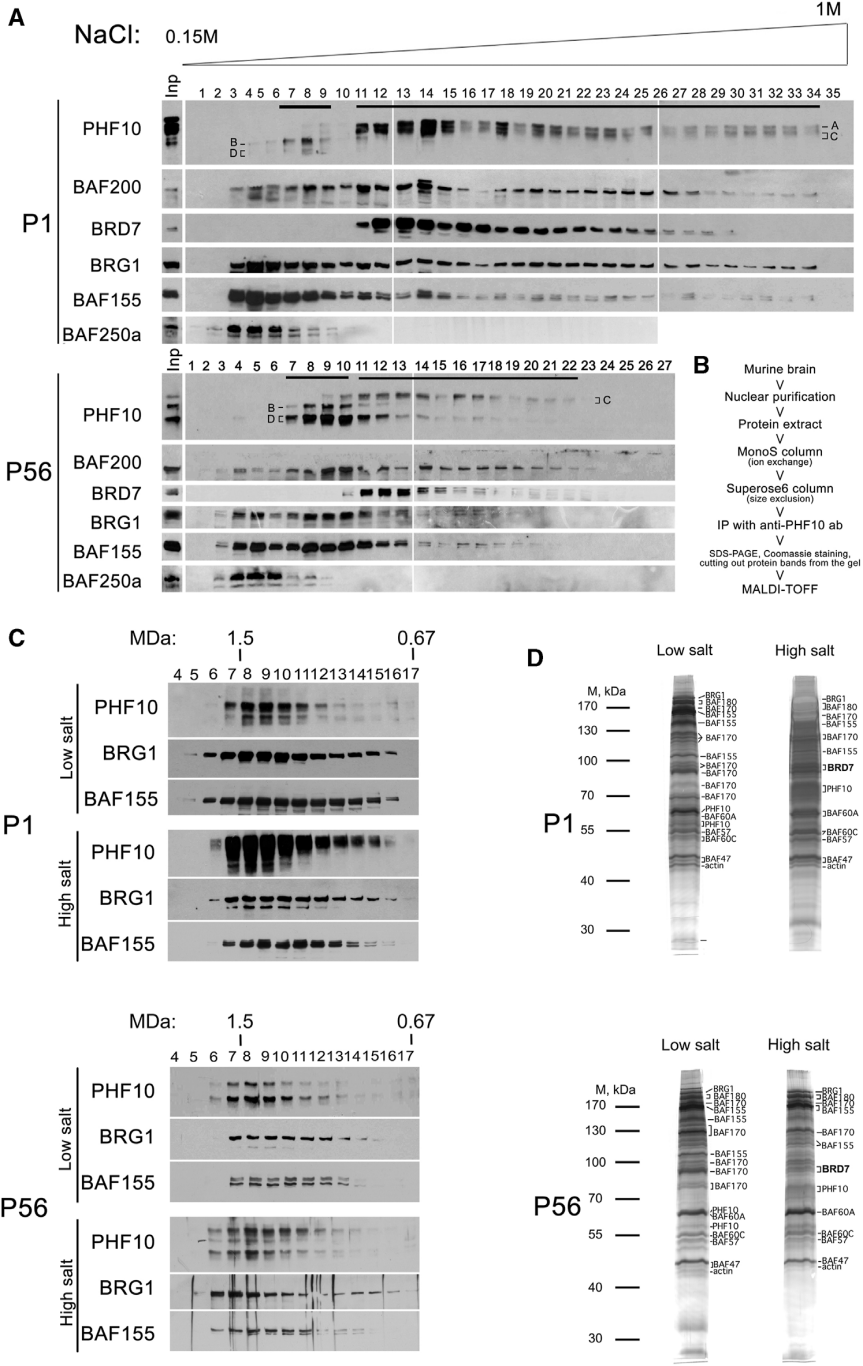
Purification of PBAF-type complexes from the P1 and P56 nuclear brain extracts. **(A)** Western blot analysis of the MonoS fractions with antibodies against different PBAF subunits. Nuclear extracts were purified from the P1 or P56 brain extracts, loaded into the MonoS cation exchange column, and eluted by a buffer containing an increasing gradient of NaCl (concentration is indicated). The proteins of each fraction were resolved *via* SDS-PAGE and analyzed for PBAF subunits. Input (Inp) is shown on the left. PHF10 isoforms are indicated. **(B)** Flowchart of the purification of the PBAF complex. **(C)** Size-exclusion chromatography of PBAF migrating in low- and high-salt MonoS fractions. The MonoS fractions containing either high- or low-salt complex (indicated by black lines on Panel 2A) were collected and loaded on the Superose 6 gel-filtration column. The proteins from Superose 6 fractions were resolved *via* SDS-PAGE and analyzed for the presence of PBAF subunits by corresponding antibodies (indicated). **(D)** Fractionation of PBAF complexes purified from the P1 and P56 extracts *via* SDS-PAGE. The PBAF-containing Superose 6 fractions from each purification were collected, and PBAF complexes were immunoprecipitated with antibodies against PHF10 bound to protein A Sepharose. The immunoprecipitates were washed and eluted from the antibodies (protein A Sepharose) using 0.1M Gly at pH = 2.5. The precipitated proteins were resolved *via* SDS-PAGE, visualized by Coomassie or silver staining, and subjected to identification as determined by trypsin digestion and MALDI-TOF analysis. The elution profiles of PBAF subunits characterized by immunoblotting after each fractionation have shown that the PBAF-type complexes kept their integrity during fractionation. The presence of BAF200 in each of the purified complexes was confirmed by Western blot.

Mouse and human PHF10 have a DPF-containing isoform and an isoform that lacks DPF (Brechalov et al., 2014; Chugunov et al., 2021). In addition, both isoforms have a truncated version without 46 N-terminal amino acids. In this study, PHF10 isoforms are designated as A, B, C, and D (Figure 1A; Supplementary Figure S1,S2; Supplementary Table S1). Here, we aimed to purify and compare PBAF from newborn and adult mouse neurons and to study PHF10A and PHF10D in PBAF at different stages of the postnatal development of the mammalian brain. The analysis of the database presented in Yao et al. (2021) has shown that the PHF10 transcription level was high in various types of neurons but was undetectable in other cells of the adult mouse brain (Figure 1B). Thus, antibodies against PHF10 were used to purify the PBAF-type complexes from the newborn (P1) and adult (P56) whole brain nuclear extracts. To eliminate the non-specifically associated proteins, the extracts were fractionated on mono S cation exchange and Superose 6 gel filtration columns, and only fractions containing the PBAF subunits were used in further purification steps (Figures 2A,B). The PBAF subunits were eluted from the mono S column as a wide peak (300 mM-1M NaCl) with an apex of approximately 350 mM NaCl during fractionation of the P1 extract (Figure 2A). Unexpectedly, we observed an additional minor peak of PBAF that was eluted with lower salt fractions, corresponding to 200–300 mM NaCl. The P56 fractions also contained two distinct peaks of PBAF, but in this case, the peak corresponding to the low-salt PBAF strongly prevailed (Figure 2A). The second peak was readily seen when fractions were stained with the antibodies against PBAF-specific subunits, PHF10 or BAF200. It can also be seen by the migration profiles of BRG1 or BAF155, although they do not give such a clear picture, as BRG1 and BAF155 are also the components of BAF (the BAF profile was indicated by its specific BAF250a subunit).

We also found that in both extracts (P1 and P56), the BRD7 subunit co-migrated with the high-salt peak, suggesting its association only with PBAF present in these fractions. Staining with antibodies against PHF10 indicated that PHF10A and PHF10D were associated with different peaks. PHF10A migrated with the high-salt peak, whereas PHF10B migrated with the low-salt peak. PHF10B and PHF10C were present in the high- and low-salt peaks, respectively. Significantly, PHF10D was dominant in the low-salt peak in the P56 extract. These results suggest the existence of a distinct type of the PBAF complex that mostly replaces canonical PBAF in differentiated neurons. We further refer to it as dcPBAF, the differentiated cell PBAF, to distinguish it from the canonical PBAF complex.

### dcPBAF does not contain BRD7

Next, the MonoS fractions from P1 and P56 extracts containing PBAF or dcPBAF were collected and fractionated on a Superose 6 size exclusion column (Figure 2C). The PBAF subunits migrated in the same Superose 6 fractions of molecular weight of approximately 1.4 MDa, which correlated with the known molecular weight of PBAF (Leschziner et al., 2005). Next, PBAF-containing fractions were collected, and the whole complex was precipitated from each pool of fractions using antibodies against PHF10, covalently bound to the resin. The precipitated proteins were eluted from the antibody-resin column, resolved on SDS-PAGE, and stained with Coomassie (Figure 2D).

The strongest Coomassie-stained bands were cut out from the gel, and the proteins were identified by the MALDI-TOF MS, which indicated that they contained mostly PBAF subunits (Supplementary Table S2). PBAF purified from P1 and P56 extracts had identical protein content, including BRG1, BAF180, BAF170, BAF155, BAF60, BAF57, BAF47, PHF10, b-actin, and BRD7 subunits that corresponded to the established protein content of PBAF (Mashtalir et al., 2018). dcPBAF purified from the P1 and P56 extracts differed from PBAF due to the absence of the BRD7 subunit.

The identified interactions were confirmed in co-immunoprecipitation experiments (Supplementary Figure S3). We have found that antibodies against PHF10 co-precipitate BRD7 from the P1 extract, which contains mostly the canonical PBAF complex. However, from the P56 extract, which contains mostly dcPBAF, the antibodies against PHF10 did not co-precipitate BRD7. These results were confirmed by reciprocal precipitation with antibodies against BRD7. As previously described, it was shown that BAF180/PBRM1 requires BRD7 and BAF200/ARID2 for association with PBAF (Mashtalir et al., 2018). We verified the association of these subunits with dcPBAF by co-immunoprecipitation (Supplementary Figure S4). The results demonstrate that BAF180/PBRM1 remains to be associated with dcPBAF in the absence of BRD7.

In summary, our results demonstrated that dcPBAF differs from canonical PBAF in proliferating cells due to the absence of the BRD7 subunit and the presence of the PHF10D isoform.

### The PHF10D isoform replaces PHF10A upon the start of the mouse neuroblast differentiation program and becomes the only PHF10 isoform present in the terminally differentiated neurons

**FIGURE 3:**
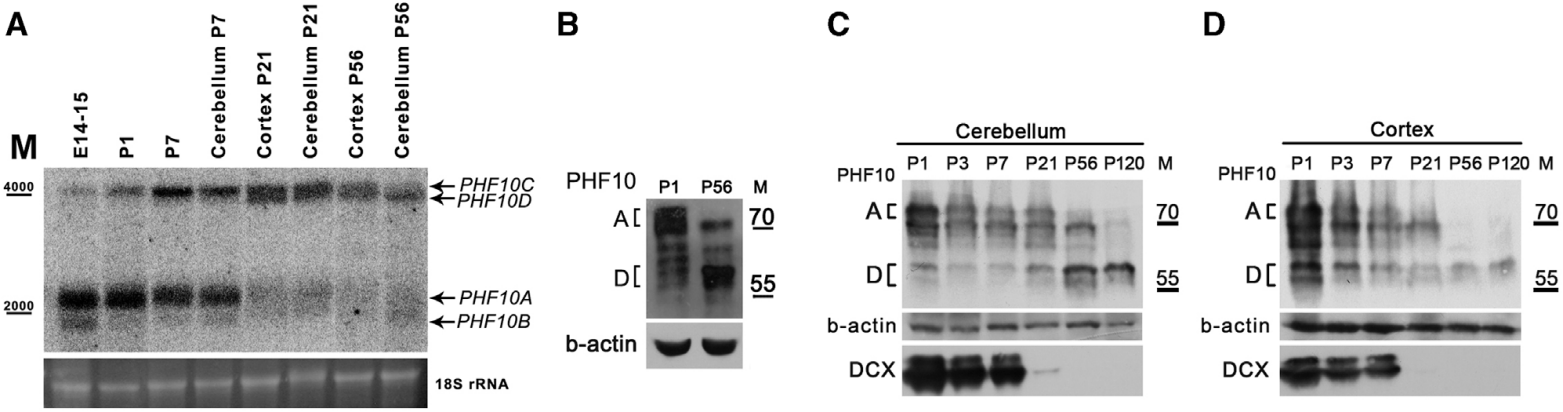
The PHF10D isoform is dominant in brain neurons of adult mice. **(A)** Northern blot analysis of PHF10 transcripts at different stages of mouse brain development. polyA RNA was purified from the whole brain extract, cortex, or cerebellum. 18S ribosomal RNA which remained in samples was used as the loading control. **(B)** and **(C)** Western Blots demonstrate the expression of PHF10A and PHF10D isoforms (indicated) at P1 and P56 stages of postnatal brain development. **(D)** Western blot demonstrating the expression of PHF10A and PHF10D isoforms (indicated) in the cerebellum and cortex at different stages of postnatal brain development. DCX was used as a marker of the exit of neurons from active divisions and b-actin as loading. It should be noted that only PHF10D remained in the completely mature neurons (p120).

We further analyzed the PHF10 expression of PHF10A and PHF10D at different stages of brain development. The Northern blot analysis has shown that the *PHF10A* transcript was dominant at the embryonic and early postnatal (P1-P7) stages but nearly completely disappeared between 7 and 23 days (Figure 3A). At the same time interval, the level of the *PHF10D* encoding transcript started to increase, and it became dominant at the later stage (P56). This result was confirmed by qPCR (Supplementary Figure S5). In line with data on transcription analysis, the Western blot indicated a significant difference in the PHF10A and PHF10D isoform expression patterns between P1 and P56 (post-natal days 1 and 56) stages (Figures 3B,C). PHF10A disappeared around 21 days of development; its expression correlates with that of doublecortin (DCX), which is the marker of young, immature neurons, indicating that PHF10 isoform switching is likely linked to neuronal maturation (Di Bella et al., 2021). PHF10D became dominant at P56, and it remained the only PHF10 isoform present at P120, which corresponds to complete neuron maturation (Hammelrath et al., 2016) (Figure 3C).

### dcPBAF localizes at the promoters of actively transcribed housekeeping and neuron-specific genes in P120 neurons

The role of PHF10A/BAF45 and the PBAF complex in the control of genes responsible for the proliferation of neural cell progenitors was shown before (Lessard et al., 2007). In this study, we aimed to characterize the genes under the control of the PHF10D and dcPBAF complex. We analyzed the genome-wide distribution of dcPBAF in neurons of adults (P120) by chromatin immunoprecipitation-coupled sequencing (ChIP-seq) using antibodies against PHF10. The chromatin was prepared from the brains of the P120 animals because at this stage, differentiated neurons contain only the dcPBAF complex, as can be seen by the high expression level of the PHF10D isoform which is a marker of dcPBAF (Figure 3C).

**FIGURE 4:**
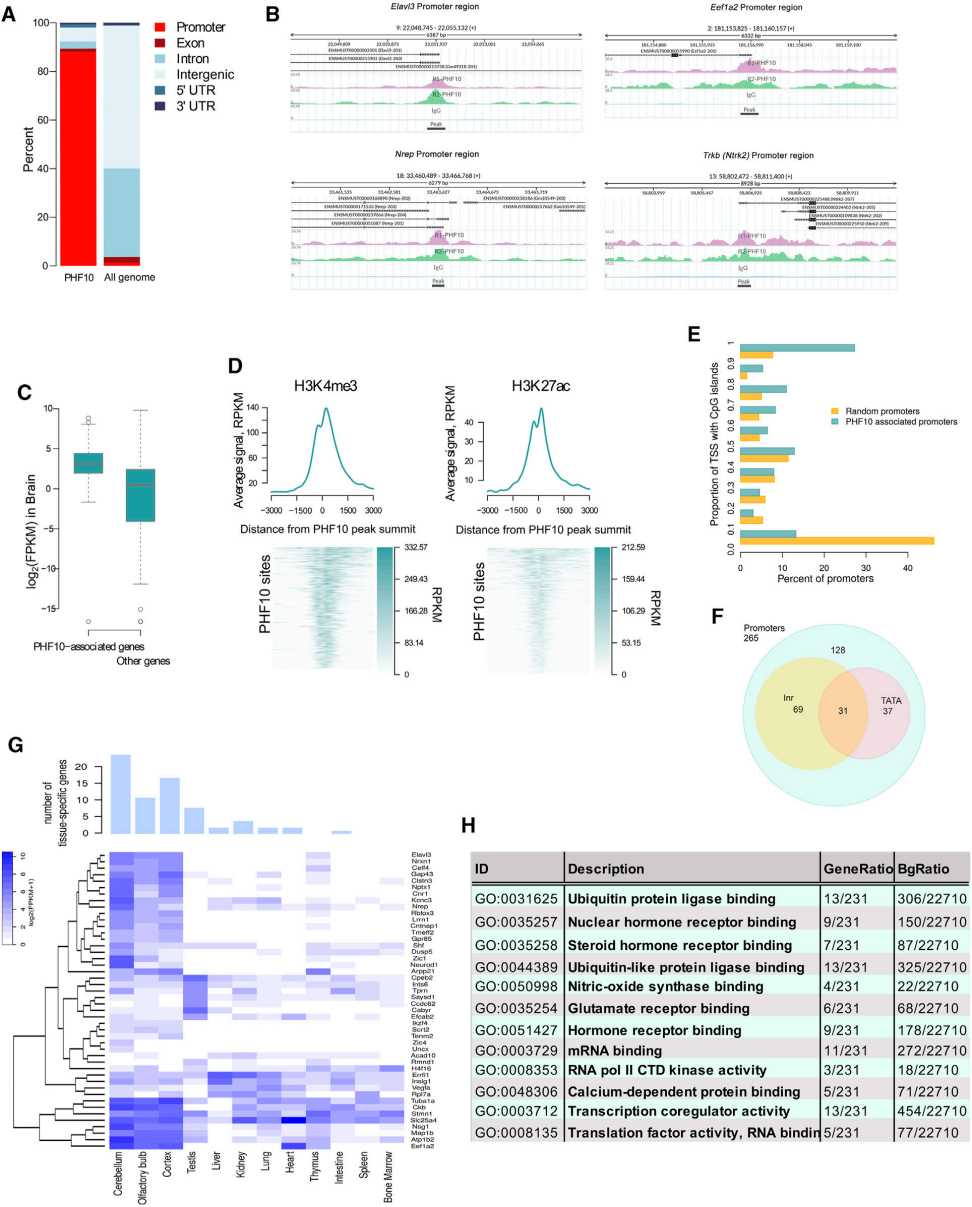
PHF10D-binding sites in the adult brain are associated with promoters of highly expressed genes. **(A)** Distribution of genomic elements in PHF10D-binding sites and in random sites distributed uniformly along the genome (N = 301). **(B)** Sequencing alignment tracks of PHF10 ChIP-seq on promoters of *Elavl3*, *Eef1a2*, *Nrep*, and *Trkb* genes; black bars depict the so-called peaks. **(C)** Average expression of genes with PHF10-binding sites in promoter regions (±200 bps from TSS) in the adult brain (Shen et al., 2012) (N = 238 genes for which the expression data were provided out of 265 PHF10-dependent genes). **(D)** Average H3K4me3 and H3K27ac ChIP-Seq signal in the mouse brain (Shen et al., 2012) in the region + -3 kb from the PHF10 peak summits. On the heatmaps, the PHF10D-binding sites are ranked according to the average value of the PHF10 signal (N = 301). The regions were oriented in such a way that the gene whose promoter is closest to the protein site has a direction from the 3′ to 5′ end. **(E)** Distribution of the proportion of TSS intersecting with CpG islands per promoter for the PHF10-dependent genes and a random gene set. The percentage of TSS containing CpG islands at the PHF10D-occupied promoters significantly exceeded that for the randomly selected promoters (Mann–Whitney test *p*-value = 2×10^-16^, panel 4D). **(F)** Numbers of PHF10-dependent genes with TATA-box and Inr elements. **(G)** Tissue-specific genes with the PHF10-dependent promoters (see Methods) (N = 46). The bar plot shows the numbers of tissue-specific genes for different tissues. The heatmap represents gene expression data (Shen et al., 2012) in different tissues. The embryonic tissues were excluded from the visualization. **(H)** Results of GO enrichment analysis for genes with PHF10 binding sites in the promoter regions (N = 254 genes for which there exists Entrez Gene ID out of 265 PHF10-dependent genes).

The analysis of the ChIP-seq results has shown that 88% (265 out of 301) of the identified PHF10D peaks overlapped with promoter regions of genes (+/-200 bp from the transcription start sites) (Figures 4A,B; Supplementary Figure S6,S7). The analysis of Mouse ENCODE data (Shen et al., 2012) has shown that PHF10D-occupied genes had a significantly higher expression level in the differentiated brain than the average (Mann–Whitney test *p*-value = 2 × 10^−16^, Figure 4C). Thus, PHF10D-containing dcPBAF is associated with actively transcribed genes. In addition, H3K4me3 and H3K27ac histone marks associated with active transcription were enriched around the PHF10D binding sites (Figure 4D), which was shown by the analysis of data on H3K4me3 and H3K27ac mark distribution on cis-regulatory sequences of the brain of adult mice (Shen et al., 2012).

The analysis of the core promoter elements of the dcPBAF-occupied genes has shown that they contained TATA-box and Inr elements, and were enriched with CpG islands (Figures 4E,F). The percentage of TSS containing CpG islands at the PHF10D-occupied promoters significantly exceeded that for the randomly selected promoters (Mann–Whitney test *p*-value = 2 × 10^−16^, Figure 4G).

An analysis of the 238 PHF10D-bound genes for which the expression values were provided (Shen et al., 2012) has shown that 75 (31.5%) of them belong to the constitutively expressed housekeeping genes according to the HRT Atlas v1.0 (Supplementary Table S3) (Hounkpe et al., 2021). The analysis of the expression values of genes occupied by PHF10D in different tissues (Shen et al., 2012) demonstrated that 46 genes (out of 238) were tissue specific. Among them, the majority were specific to the brain (Figure 4G).

The gene set enrichment analysis (GSEA) using the GO database identified significantly enriched pathways among the dcPBAF target genes (FDR<0.05) (Figure 4H). These were the neuron-specific pathways, like hormone and glutamate receptor binding, nitric oxide synthase, calcium-dependent protein binding, and neuronal transcription regulation activity. There were also sets of genes regulating general cellular processes and reflecting the activity of housekeeping genes, like protein biosynthesis and degradation, ubiquitin protein ligase binding, transcription co-regulator activity, or mRNA binding.

### The PHF10D isoform is dominant in human brain neurons upon differentiation

**FIGURE 5:**
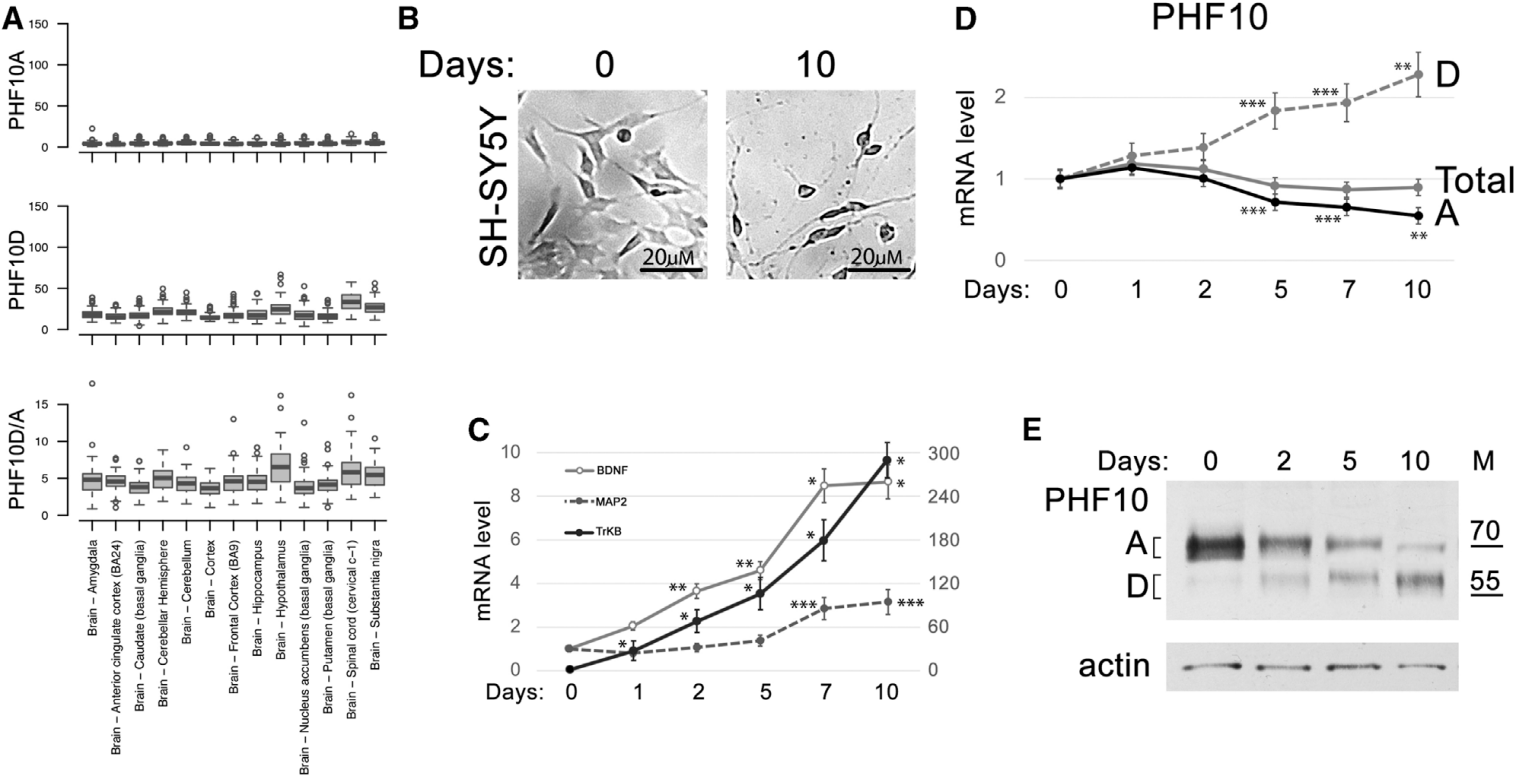
PHF10D isoform becomes dominant in the developed human neuronal tissues. **(A)** Expression of PHF10A and PHF10D isoforms (TPM) and their relative ratio (D/A) in different parts of the human brain according to the GTEx project data (GTEx Consortium, 2013). **(B)** SH-SY5Y differentiation after ATRA and BDNF treatment for 10 days (large view, Supplementary Figure S9). **(C)** Expression of *BDNF*, *MAP2* (changing according to the right scale), and *TrkB* (changing according to the left scale) marker genes increased during SH-SY5Y differentiation. **(D)** The levels of total PHF10 transcript (PHF10-total) and transcripts encoding PHF10A and PHF10D isoforms at different timepoints (days) after the start of differentiation of the SH-SY5Y neuronal cells. **(C, D)** Values represent the mean ± SD of three independent experiments and were normalized to *RPLP0* expression. The *Y*-axis indicates mRNA fold change. Statistically significant differences are marked as *p < 0.0005, **p < 0.005, and ***p < 0.05 compared to the control (0 h) (two-way ANOVA with Holm–Sidak’s multiple comparison test). **(E)** Western blot demonstrating the expression of PHF10A and PHF10D isoforms at different timepoints (days) after the start of differentiation of the SH-SY5Y neuronal cells. Actin is used as a loading control.

PHF10 isoforms are highly conserved between mice and humans (Brechalov et al., 2014; Chugunov et al., 2021). Human and mouse isoforms have the same molecular weight and differ by only several amino acid substitutions. The database analysis has shown that similar to that found in mice, the PHF10 transcription level was high in various types of neurons but was close to background in the non-neuronal cells of the adult brain (Supplementary Figure S8). The analysis of Genotype-Tissue Expression (GTEx) project data indicated that the PHF10D isoforms strongly prevailed over PHF10A in different zones of the adult human brain (Figure 5A).

To further study the PHF10A and PHF10D isoform expression during neuronal differentiation, the human SH-SY5Y neuroblastoma cell culture was used. Cells were treated with ATRA and BDNF according to the described protocol (Kovalevich et al., 2013) which led to their partial differentiation. It includes a decrease in the proliferation, the acquisition of neuron-specific morphology, and an increase in the transcription of genes specific for differentiated neurons, like *BDNF*, *MAP2*, and *TrkB* (Figures 5B,C; Supplementary Figure S9). We found that the level of the *PHF10D* transcript started to increase after the induction of differentiation and became two-fold higher by day 10, whereas the level of the *PHF10A* transcript decreased (Figure 5D). Western blot analysis has shown that the PHF10A isoform disappeared by the 10th day of differentiation. On the contrary, the level of the PHF10D isoform, which was very low at the start of differentiation, increased after day 5 (Figure 5E). Thus, similar to the observations made in mice, the replacement of PHF10A by PHF10D expression coincides with the maturation of neural cells.

### The PHF10D isoform is essential for the transcription of human neuron-specific genes

**FIGURE 6:**
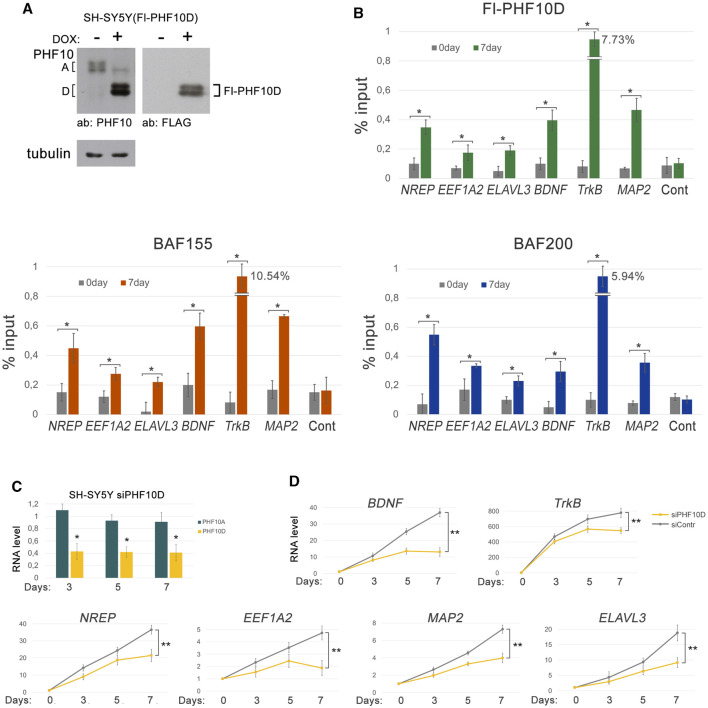
The PHF10D isoform controls the expression of the neuron-specific genes in SH-SY5Y. **(A)** The expression levels of the FLAG-tagged PHF10D isoform in the SH-SY5Y cells before and following the induction by doxycycline, verified by Western blot. **(B)** The levels of the Fl-PHF10D isoform, BAF155, and BAF200 on promoters of the neuron-specific genes, *TrkB*, *MAP2*, *BDNF*, *EEF1A2*, *NREP*, and *ELAVL3*, before and following differentiation of the SH-SY5Y cells, verified by ChIP. The level of PHF10D at the non-coding region (see Materials and Methods) was used as a control. **(C)** The level of PHF10D and PHF10A transcripts in the SH-SY5Y cells with knockdown of PHF10D relative to the control cells treated with siControl at different timepoints following the start of differentiation. The level of transcripts in control cells was taken as one. Knockdown was performed on the next day after the addition of ATRA, and then repeated on the fourth and sixth days of differentiation. **(D)** The levels of transcription of the *TrkB*, *MAP2*, *BDNF*, *EEF1A2*, *NREP*, and *ELAVL3* neuron-specific genes in the SH-SY5Y cells with knockdown of PHF10D and in the control SH-SY5Y at different timepoints following the start of differentiation. The values represent the mean ± SD from three independent experiments. Statistically significant differences are marked as *p < 0.005 and **p < 0.0005 for pairs of compared data (two-way ANOVA with Holm–Sidak’s multiple comparison test).

Finally, we addressed the role of the PHF10D isoform in the maintenance of transcription of genes specific for differentiated human SH-SY5Y cells like *TrkB*, *MAP2*, *BDNF*, *NREP*, *EEF1A2*, and *ELAVL3*. The mouse homologs of these genes have PHF10D on their promoters, as shown in ChIP-seq (Figure 4B, Supplementary Figure S7). To verify their promoter occupation by PHF10D in human cells, an Fl-PHF10D isoform under the doxycycline (DOX)-inducible promoter was expressed in SH-SY5Y (Figure 6A). The Fl-PHF10D expression was induced on the next day after ATRA treatment and maintained throughout the differentiation time course. As was verified by ChIP with anti-FLAG antibodies, the Fl-PHF10D isoform as well as BAF155 and BAF200 subunits of PBAF efficiently bound the promoters of all studied genes following differentiation (Figure 6B).

Next, the effect of a knockdown of PHF10D in SH-SY5Y cells on transcription of *TrkB*, *MAP2*, *BDNF*, *NREP*, *EEF1A2*, and *ELAVL3* genes was studied. The level of the PHF10D transcripts decreased two-fold, which confirmed the efficiency of the knockdown (Figure 6C). The knockdown of PHF10D led to a significant decrease in the transcription of all studied genes, indicating that PHF10D maintains neuron-specific gene transcription (Figure 6D).

## Discussion

**FIGURE 7:**
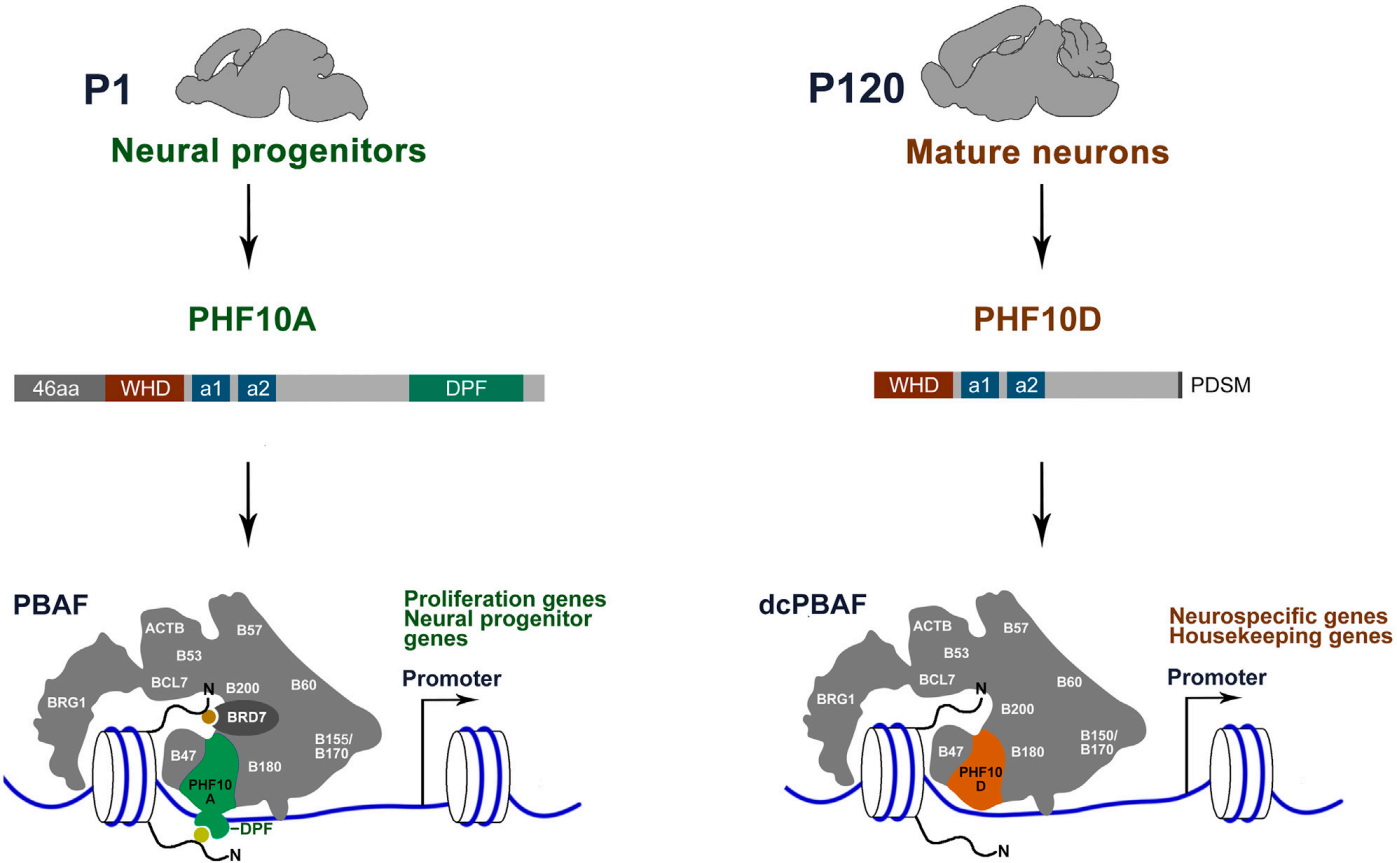
Distinct type of PBAF complex function in mature neurons of adult mice. The canonical PBAF contains the PHF10A/BAF45a isoform of PHF10 which has previously been shown to be required for the proliferation of neural stem and progenitor cells (Lessard et al., 2007). PBAF contains the PHF10A isoform with the DPF domain and BRD7 with the BD for histone modification (yellow and orange circles) binding. dcPBAF of terminally differentiated neurons occupies promoters of the neuron-specific and housekeeping genes. dcPBAF contains the PHF10D isoform and does not contain the BRD7-specific PBAF subunit.

Here, we show that neurons in the adult mouse brain contain a distinct version of the PBAF complex (dcPBAF) (Figure 7). Whereas canonical PBAF is dominant in proliferating neuronal cells and immature neurons, the level of dcPBAF starts to grow following the beginning of differentiation, and it replaces canonical PBAF in terminally differentiated mature neurons. dcPBAF lacks the BRD7 subunit and includes PHF10D, the specific isoform of the PHF10 subunit that does not have the 46N-terminal amino acids and C-terminal DPF domain (Figure 7). dcPBAF binds the promoters of highly expressed neuron-specific and housekeeping genes.

The PHF10 PBAF subunit is highly expressed in mouse brain neurons. Two isoforms of PHF10, PHF10A, and PHF10D, are associated with two different states of the neuronal cell development program: immature neuroblasts and terminally differentiated mature neurons, respectively. The PHF10A/BAF45a isoform promotes the proliferation of neuroblasts (Lessard et al., 2007). Our study points to the importance of the DPF-lacking PHF10D isoform in the later stages of development. Its expression level increases as mouse neurons start the differentiation program. The PHF10D isoform is present on promoters of actively transcribed neuron-specific genes in adult mice. In human SH-SY5Y cells, PHF10D occupies promoters of neuron-specific genes following the start of differentiation and is essential for their high transcription level. The same replacement of PHF10A with PHF10D was previously shown to occur during myeloid differentiation (Viryasova et al., 2019), suggesting that dcPBAF-containing PHF10D functions in various types of terminally differentiated mammalian cells. In addition to mammals, DPF-lacking isoforms were also found in several other vertebrate species (Brechalov et al., 2014; Chugunov et al., 2021). The conserved structure of the PHF10 isoforms in mammalian species indicates that the replacement of DPF-containing by the DPF-lacking isoforms in the PBAF complex during differentiation may be a general mechanism (Sheynov et al., 2019).

The role of the replacement of PHF10A by PHF10D in terminally differentiated cells, for example, neutrophils and mature neurons, is not clear yet. However, previously we have demonstrated that PHF10A is a strong transcriptional co-activator, whereas PHF10D does not activate transcription (Brechalov et al., 2014). PHF10A/BAF45a was found to occupy the promoters of several components of the Notch signaling pathways in mouse neural progenitor cells (Lessard et al., 2007). It is essential for the maintenance of adult mouse hematopoietic stem cells (Krasteva et al., 2017) and for the proliferation of human myeloid progenitors (Viryasova et al., 2019). One may assume that PHDF10A is specifically important for the transcription activation of genes responsible for the proliferation of immature precursors in different mammalian tissues.

Our data demonstrate that dcPBAF does not contain Brd7 which is an important subunit of PBAF known as a tumor suppressor that inhibits growth and metastasis and initiates apoptosis in cancer cells (Yu et al., 2016; Niu et al., 2020; Chen et al., 2021). BRD7 and PHF10 are PBAF-specific subunits that physically interact with each other, forming together with PBRM1 the histone tail interaction submodule of PBAF (Yuan et al., 2022). The BD of BRD7 has been shown to possess a high affinity for the H3K14 active chromatin mark (Peng et al., 2006; Sun et al., 2007). The PHF10A DPF domain as predicted has a high affinity for H3K14ac and differentiates this mark from H3K4me3 (Chugunov et al., 2021). Of the six DPFs present in mammalian proteins, four DPF1-3 and PHF10 are members of the BAF and PBAF chromatin-remodeling complexes, respectively. Some of them have been shown to be involved in interactions with histone modifications (Local et al., 2018; Chugunov et al., 2021). It can be assumed that the absence of PHF10A and BRD7 subunits affects the specificity of PBAF–chromatin interactions and directs dcPBAF to a program that is specific for differentiated cells.

## Materials and methods

### Mice

We used 1- to 120-day-old male C57BL/6J mice (Jackson Laboratory, United States). Animals were kept in identical conditions (a standard light- and temperature-controlled environment, 12-h light/dark cycle; light on at 7:00 AM; 21°C, with access to food and water *ad libitum*) at NRC Kurchatov Institute (Moscow, Russia).

For protein extracts or RNA preparation, the whole brains, cortex, and cerebellums were isolated with surgical scissors from the decapitated mice at the age needed. Then, the brain/cerebellum was snap-frozen in liquid nitrogen or placed on ice and homogenized with sterile plastic pestles in a suitable buffer.

### Cells, differentiation, and knock-down

The human neuroblastoma SH-SY5Y cell line (from American Type Culture Collection, Manassas, VA) was propagated in the Dulbecco modified Eagle’s medium (DMEM; PanEco, Russia) with 10% heat-inactivated fetal bovine serum (hiFBS) (HyClone, Logan, UT) supplemented with 2 mM *L*-glutamine (Merck) and penicillin/streptomycin at 37°C with 5% CO_2_ in a humidified atmosphere.

Differentiation was performed according to the published protocol (Kovalevich et al., 2013; Moysenovich et al., 2020). For differentiation, cells were seeded at 100,000 per one 35-mm Petri dish. The next day, the media were replaced by differentiation media 1 (DMEM; 2.5% hiFBS; 2 mM *L*-glutamine; penicillin/streptomycin; and 10 μM all-trans retinoic acid (ATRA) (Sigma)). Three days later, the media were replaced by differentiation media 2 (neurobasal media (Gibco); 1x B27 (Invitrogen); 2 mM GlutamaxI (Invitrogen); 10 μM ATRA (Sigma); and 50 ng/mL BDNF (Novus Bio/R&D Systems, #248-BD)), and cells were incubated for 7 days.

The knockdown of PHF10D was performed in the SH-SY5Y cells on the first day (before ATRA addition), fourth day, and sixth day of differentiation, by two different siRNA simultaneously, or control siRNA (Supplementary Table S3). Metafecten Pro (Biontex) and siRNA (sequences are listed in Supplementary Table S4) were mixed in OptiMEM (Gibco) according to the manufacturer’s protocol, incubated for 15 min at room temperature, and added to the cells. After 12 h, the media were replaced by differentiation media 1 or 2. Cells were harvested according to the time course.

### Western blot analysis

For Western blot analysis, the whole brain, cortex, or cerebellum was homogenized on ice in 10 volumes (W:V) of RIPA buffer; 50 mM Tris-HCl pH 7.4; 1% NP-40; 0.5% Na deoxycholate; 0.1% SDS; 150 mM NaCl; 2 mM EDTA; protease inhibitor cocktail (PIC, Sigma); and phosphatase inhibitor cocktail (PhIC, Sigma), and then 4× Laemmli buffer (200 mM Tris-HCl pH = 6.8; 4% SDS; 40% glycerol; 0.01% bromophenol blue; and 100 mM DTT) was added, boiled for 10 min, and snap-frozen in liquid nitrogen for further Western blotting analysis.

The SH-SY5Y cells were scrapped from the 60-mm Petri dishes, washed with PBS supplemented with PIC, centrifuged at 4°C, 2.5 krpm for 5 min, and resuspended in 10V of RIPA buffer, incubated for 10 min on ice, and centrifuged at 13.2 krpm at 4°C for 10 min. Protein concentration was measured using the Qubit Protein Assay Kit (Thermo Fisher Sci.). Then, 4× Laemmli buffer was added, probes were boiled for 10 min, and Western blotting was performed.

Affinity-purified polyclonal antibodies against the PHF10, BAF155, BAF200, BRG1, and BAF250a subunits were described previously (Brechalov et al., 2014; Tatarskiy et al., 2017). The polyclonal rabbit anti-PHF10 antibodies were raised against 238–261 amino acids of PHF10 (NP_060758.2). These antibodies recognize all PHF10 isoforms. The antibody against FLAG (M2) was obtained from Sigma; the antibody against doublecortin (ab235153) was obtained from Abcam; and antibodies against tubulin (9F3), GAPDH (14C10), and b-actin (D6A8) were obtained from Cell Signaling. HPR-conjugated anti-rabbit IgG and HPR-conjugated anti-mouse goat IgG were obtained from DHGB, and HRP-conjugated anti-goat antibodies (AP108P) were obtained from Sigma-Aldrich.

### Cloning and stable line generation

For the Fl-PHF10D-pSLIK expression vector, Fl-PHF10D cDNA was cloned from the previously described Fl-PHF10D-pcDNA (Cell Cycle, Tatarskiy) into the pSLIK plasmid (Addgene #25735 (Shin et al., 2006)) and modified by Soshnikova et al. (2021). The lentiviral particles were produced using pCMV-VSV-G (Addgene #8454 (Stewart et al., 2003)) and pCMV-dR8.2 (Addgene #8455 (Stewart et al., 2003)) plasmids, according to the previously described protocol (Soshnikova et al., 2021). The SH-SY5Y cells of the eighth passage were treated with lentiviral particles, and the next day, the media were replaced with the media containing 0.5 μg/mL G418.

### Nuclear extract preparation

Whole brains from P1 (1 g) or P120 (1 g) mice were homogenized in five volumes of NU-1 (15 mM HEPES-KOH pH = 7.6, 10 mM KCl, 5 mM MgCl_2_, 0.1 mM EDTA, 0.5 mM EGTA, 1 mM DTT, and 0.35 M sucrose) supplemented with PIC (Roche) and PhIC (Sigma) on ice in Dounce loose (Millipore), and samples were incubated for 10 min on ice. After centrifugation at 4°C for 5 min at 100 g, the supernatant was transferred into new tubes and centrifuged at 4°C for 10 min at 2 kg. The pellet was washed with five volumes of NU-1 and centrifuged again in the same conditions. Next, the pellet was resuspended in an equal volume of NUS-1 (NU-1 with an additional 0.8M sucrose), gently laid on an NUS-1 cushion, and centrifuged at 4°C for 20 min at 3.5 kg. Nuclei in the pellet were resuspended in NU-2 (15 mM HEPES-KOH pH = 7.6, 11 mM KCl, 5 mM MgCl_2_, 0,1 EDTA, and 1 mM DTT), supplemented with PIC and PhIC, and transferred in Dounce tight for nuclear destruction. 1/10 V NaCl (5M) and Triton X-100 up to 0.3% were added, mixed, and incubated for 15 minutes on ice. In the last step, the solution was ultracentrifuged at 4°C for 1 hour at 55 kg.

### Purification of PHF10-containing multiprotein complexes

The nuclear extracts from the P1 and P56 whole mouse brains were used for the PHF10-containing complex purification, according to the flowchart (Figure 2B). The columns of MonoS HR 16/10, and Superose 6 HR 10/30 (GE Healthcare) were equilibrated with the HEMG buffer (25 mM HEPES-KOH at pH 7.6, 12.5 mM MgCl_2_, 0.1 mM EDTA, 10% glycerol, and 1 mM DTT) containing 150 mM NaCl (HEMG-150). Elution from MonoS HR 16/10 was performed by the gradient of increasing NaCl concentration, up to 1M (HEMG-1000). The immunoaffinity purification was performed on a column with immobilized anti-PHF10 antibodies, with the unbound protein being washed out with HEMG-1000 buffer containing 0.1% NP-40. HEMG-150 with 0.1% Nonidet P-40 was used for loading, HEMG-1000 with 0.1% Nonidet P-40 was used for washing, and 0.1 M glycine (pH 2.5) was used for elution. The eluted proteins were resolved *via* SDS/PAGE and visualized by silver staining. The protein bands were cut out and subjected to in-gel trypsin digestion. MALDI-TOF MS was performed using an Ultraflex II mass spectrometer (Bruker Daltonics). Protein spectra were internally calibrated using trypsin autolysis products, and the resulting peptide weights were searched against the non-redundant database maintained by the National Center for Biotechnology Information, using the MASCOT search engine. The Superose 6 column was calibrated with an HMW Calibration Kit (GE Healthcare). The void volume of the column was 7.0 mL, and the volume of each fraction was 0.5 mL.

### Gene expression analysis

RNA was isolated from 3 × 10^6^ SH-SY5Y cells using TRI Reagent (MRC) according to the manufacturer’s protocol. The synthesis of cDNA was performed with an oligo (dT) primer and MMLV reverse transcriptase (Thermo Fisher Scientific). PCR primers are listed in Supplementary Table S5. The values were normalized to the *RPLP0* housekeeping gene. At least three independent experiments were performed; values are presented as mean ± SD. Statistical analysis was performed using a two-way ANOVA with Holm-Sidak’s multiple comparison test and GraphPad Prism 6 software. *p-*values <0.05 were considered significant. More details are provided in Figure legends.

### Northern blot analysis

Total RNA was isolated from 0.1 g of material ground with TRI Reagent in liquid nitrogen with a mortar and pestle. Then, the mRNA fraction was enriched with the Oligotex mRNA Kit (Qiagen). mRNA (1 mkg per lane) was mixed with the loading buffer (50xFGRB (1 M MOPS pH 7.0 and 0.5 M AcONa x 3H_2_O), 12.3 M formaldehyde, and 1 M formamide), heated at 65°C for 15 min, and immediately placed on ice. The formaldehyde electrophoresis in running buffer (1xFGRB, 2.2 M formaldehyde) was performed, and then the gel was equilibrated in 20x SSC buffer (3 M NaCl and 0.3 M Na_3_C_6_H_5_O_7_ x 5.5 H_2_O). The mRNA transfer on the Hybond-N membrane (Amersham) was also performed in 20x SSC buffer with a filter paper stack overnight, and then the mRNA was crosslinked by Stratalinker 1800 with UV light. Membrane was hybridized at 50°C overnight in HSB(f+) buffer (7% SDS, 50% formamide, 5x SSC, 50 mM NaP, 0.1% N-Lauroylsarcosine Na, and 50 μg/mL herring sperm DNA) with a DNA probe against all PHF10 isoforms labeled by alpha [32P] dATP and then washed twice with 1x SSC buffer and 0.3x SSC buffer. Development was performed using Cyclone (Packard).

### ChIP and library preparation

For chromatin immunoprecipitation, whole brains from P120 mice were homogenized in ten volumes of PBS supplemented with PIC (Roche) and PhIC (Sigma) on ice in Dounce loose (Millipore). Then, formaldehyde was added up to 1%, and samples were incubated for 10 min on ice. After centrifugation at 4°C for 10 min, 1000 g samples were washed three times with the same cold PBS and then resuspended in 1 mL of the sonication buffer (50 mM HEPES-KOH pH 7.9; 140 mM NaCl; 1 mM EDTA; 1% Triton X-100; 0.1% Na deoxycholate; and 0.1% SDS) supplemented with PIC and PhIC in Dounce tight (Millipore) for nuclear destruction. The chromatin was sheared to ∼500 bp by sonication at Covaris and precipitated by 10 µL anti-PHF10 antibodies or 1 µg anti-Flag M2 antibodies and 1 µg control rabbit IgG (Invitrogen) on Mab-selected beads (GE Health). Then, beads were washed with sonication buffer, Wash buffer A (sonication buffer with 500 mM NaCl), Wash Buffer B (20 mM Tris-HCl (pH 8.0), 1 mM EDTA, 250 mM LiCl, 0.5% NP-40, and 0.5% deoxycholate Na), and subsequently, TE. DNA was eluted by elution buffer (50 mM Tris-HCl (pH 8.0), 1% SDS, and 1 mM EDTA) and treated with RNase A for 30 min and protease K (Thermo Fisher Scientific) overnight, incubated at 65°C for 6 h, and extracted using the phenol-chloroform method. DNA from SH-SY5Y was dissolved in 100 μL TE buffer, and then qPCR was performed with primers listed in Supplementary Table S6.

For NGS library preparation, 30 ng of DNA and the NEBNext Ultra II DNA Library Prep Kit for Illumina were used according to the manufacturer’s protocol. Adaptor ligation was performed using the NEBNext Multiplex Oligos for Illumina (Index Primer Set #1) (NEB). The quality of the DNA was analyzed with Bioanalyzer DNA 1000 Chip Kits (Agilent). DNA yields were assessed using Qubit assay kits (Thermo Fisher Scientific). Libraries were sequenced on an Illumina NovaSeq 6000 with single-strand 100-bp-length reads. Approximately 3 × 10^7^ reads were obtained per sample.

### Quantification of ChIP results

The raw data were represented as single-end reads from two biological replicates. Raw reads are deposited under accession number GSE199351 (reviewer token: uxyhwcseltcplqh). The average read length was 100 bp. Adapters, poly-N, poly-A, and low-quality read ends were removed using Cutadapt software (Martin, 2011) (the quality threshold was set to 20, and reads with a length less than 20 bp after trimming were discarded). The remaining reads were aligned to build version mm10 (GRCm38, Ensembl release) of the *Mus musculus* genome using Bowtie version 1 (Langmead et al., 2009) (with enabling options -best, -strata, and -tryhard). Only reads that aligned exactly once were passed through for further analysis. PCR duplication artifacts were removed using the Picard MarkDuplicates function (http://broadinstitute.github.io/picard/). In addition, peaks overlapping with blacklist regions were discarded (https://sites.google.com/site/anshulkundaje/projects/blacklists). Peak calling was performed using MACS version 2 against pre-immune control (Zhang et al., 2008), with the suppression of the model built and the assignment of the read extension length to 200 bps. Peaks with a MACS2 *p-*value less than 1 × 10^−2^ were passed to the IDR pipeline to access the reproducibility of ChIP-seq replicates (https://sites.google.com/site/anshulkundaje/projects/idr). The IDR *p*-value threshold was set to 0.05. As the reproducibility was good according to IDR (rescue ratio = 1.05; self-consistency ratio = 1.15), we formed the final set of PHF10 sites by combining the peaks obtained for each replicate with a MACS2 *p*-value less than 1 × 10^−5^. Intersecting peaks were merged during this procedure. Examples of PHF10 peaks from both replicates and IgG control are provided in Supplementary Figure S9. ChIP-seq coverage tracks for H3K4me3 and H3K27ac in the brain were obtained by analyzing previously published data (GSM722665 and GSM851271) (Shen et al., 2012). Preprocessing was performed as previously described; a coverage track was made using the deepTools (Ramírez et al., 2014) bamCoverage function with a bin width of 100 bp and RPKM normalization.

Further analysis was performed in R version 4.0.3 (Core Team and Others, 2013). The distribution of peaks between genome elements analyses was performed using ChIPseeker version 1.26.0 (Yu et al., 2015) (promoter regions were defined as ±200 bp from TSS). GO enrichment analysis of PHF10 sites was made using clusterProfiler (version 3.18.0) function enrichGO (Yu et al., 2012).

The number of PHF10-dependent promoters containing specific promoter elements (TATA-box and Inr) was obtained from the EPD database (Dreos et al., 2015). CpG islands containing TSSs were identified by intersecting published CpG island (Gardiner-Garden and Frommer, 1987) track with TSS regions (±100 bp) of PHF10-dependent promoters. The expression of genes whose promoters were associated with PHF10-binding sites in *Mus musculus* tissues was obtained from previously published data (Shen et al., 2012) using TissueEnrich R package version 1.10.0 (Jain and Tuteja, 2018) (17 tissues; among them, 12 are adult tissues). Tissue-specific genes were identified using the same package and defined as genes belonging to one of three categories: tissue enriched (FPKM>1 and at least five-fold higher expression levels in a specific tissue than others), group enriched (FPKM>1 and at least five-fold higher expression levels in a group of 2–7 tissues than others), or tissue enhanced (FPKM>1 and at least five-fold higher expression levels in a specific tissue compared to average levels in all others) (Jain and Tuteja, 2018). Housekeeping genes were determined using HRT Atlas v1.0 (Hounkpe et al., 2021).

## Data Availability

The datasets presented in this study can be found in online repositories. The names of the repository/repositories and accession number(s) can be found in the article/Supplementary Material.
