# Supplementary material for: A novel chromatin-remodeling complex variant, dcPBAF, is involved in maintaining transcription in differentiated neurons
Source: Front Cell Dev Biol. 2023 Nov 14;11:1271598. doi: 10.3389/fcell.2023.1271598 (PMC10682186; doi:10.3389/fcell.2023.1271598)
Supplement: Supplementary file 1 [file Presentation1.pdf]

Supplementary

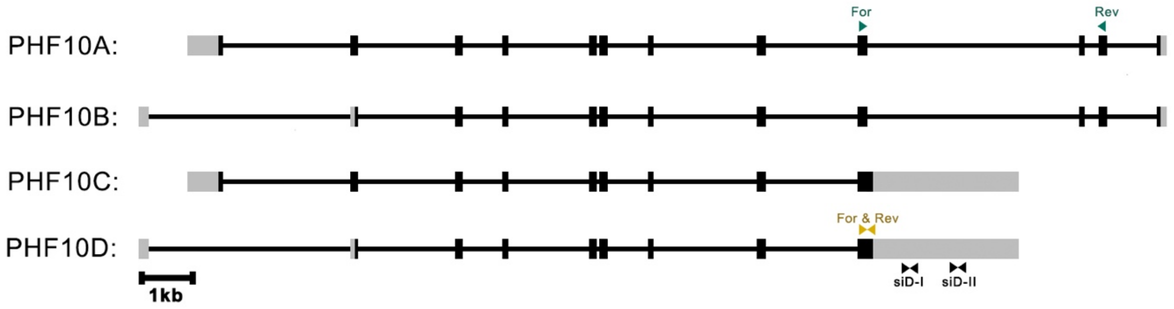

Figure S1. Schematic representation of PHF10 transcripts. The coding regions are indicated by black and the noncoding regions by gray boxes. Primers for PHF10A measurement depicted by dark-green arrows and primers for PHF10D measurement depicted by yellow arrows (sequences of primers are at the Table S5). Also siRNA-I and siRNA-II for PHF10D knockdown depicted black arrows (sequences of siRNA's are at the Table S4).

Table S1.
The human and murine PHF10 mRNAs and protein isoforms present in the NCBI databases.

Species	Isoform name	other name	transcript ID NCBI	transcript ID Length	protein ID NCBI	protein ID Length
H. sapiens	**PHF10A**	PHF10-Pl	NM_018288.4	2167bp	NP_060758.2	498aa
	**PHF10B**	PHF10-Ps	KC_839988.1	1651bp	AGQ_46690.1	451aa
	**PHF10C**	PHF10-Sl	KC_839989.1	4395bp	AGQ_46691.1	377aa
	**PHF10D**	PHF10-Ss	KC_839990.1	3878bp	AGQ_46692.1	330aa
M. musculus	**Phf10A**	Phf10-Pl	NM_024250	1665bp	NP_077212.3	497aa
	**Phf10C**	Phf10-Sl	NM_001360983	3992bp	NP_001347912.1	376 aa

The analysis of mouse and human PHF10 transcripts has shown that like other mammals they have the splice variants encoding DPF containing isoform and DPF lacking isoform (Brechalov et al., 2014, Chugunov et. al., 2021). The human PHF10 transcripts were precisely mapped (Brechalov et al., 2014). The Northern blot analysis demonstrates that mouse and human have the same four PHF10 transcripts of the similar length. In Database are annotated mouse transcripts encoding long PHF10 isoforms: Phf10A (NM_024250 - 1665 nt, NP_077212.3 - 497 aa) and Phf10C (NM_001360983 - 3992 nt, NP_001347912.1 - 376 aa).

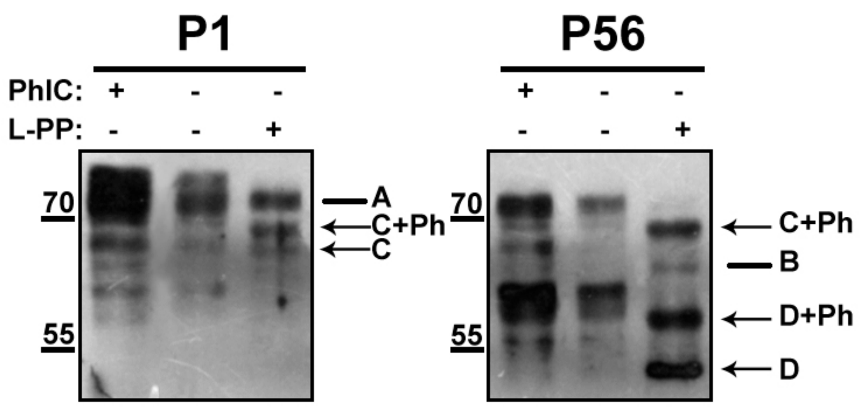

Figure S2. PHF10 isoforms undergo multiple phosphorylation as was confirmed on brain extracts treated with the Lambda-phosphatase (L-PP).
PHF10 is comprised of four major bands, corresponding to the described PHF10 isoforms, and several additional bands which appeared due to multiple phosphorylation the PHF10 isoforms (depicted as +Ph). Note that on Figure 1 and after, the PHF10 isoforms are indicated according to the analysis of their electrophoretic mobility performed previously (Tatarskiy et al., 2017). Each PHF10 isoform has its own specific phosphorylation pattern, due to the difference in their structure. PhIC + – nuclear extract prepared with Phosphatase inhibitor cocktail addition. L-PP + – nuclear extract prepared with Lambda phosphatase addition.

Table S2. Proteins co-precipitated by anti-PHF10 antibodies were eluted and identified by MALDI-TOFF.

Low salt PBAF - P56	High salt PBAF - P56
BRG1	BRG1+BAF180
BAF180	BAF180
BAF170	BAF170
BAF155	BAF180+BAF155
TAF4A	BAF180+BAF170
BAF170	BAF170
BAF170	BAF170
BAF170	BRD7
TAF5	PHF10
BAF170	BAF60A
TAF6	BAF60C
BAF170	BAF57
PHF10	BAF47
keratin	BAF47
BAF60A	BAF47
BAF60C	actin
BAF57	TfII-I
BAF47	Ilf2
BAF47	
actin	

Ilf2<br>Pcsk6<br>TAF9	

Low salt PBAF – P1	Copurified proteins	High salt PBAF - P1	Copurified proteins
Galnt2		BRG1	BAF190B
BRG1		BAF180	Ccdc88C
BAF180	Map6	BAF170	Rock1
BAF170	Dna2	BAF155	
BAF170	Iqgap2, Znf407	BAF170	Vav3, Lrrtm2
Pacs1		BAF155	PkC
BAF170		Rasal3	Rock1, Dna2
PHF10	Tsga10	BAF155+BAF170	Rock1 Zc3h12[illegible]
BAF170	Adamts15,Pibf1	BRD7	Rnf213
TAF5	ccp110	PHF10	Ercc1, Haus1
IgG	Ddb2	PHF10	
TAF6		BAF60A	D15Kzl
BAF170	Tin2, Rcor2	BAF60C	Zfp709l1
PHF10		BAF57+BAF60C	
BAF60A	Wdr37	BAF57	
Wdr37	Znf592	BAF47	
BAF57		BAF47	
BAF57	Samd3	actin	
BAF47			
Cnot2			
BAF47	Nr1d2		
actin			
Sh3d19	Skor1		
TAF9			

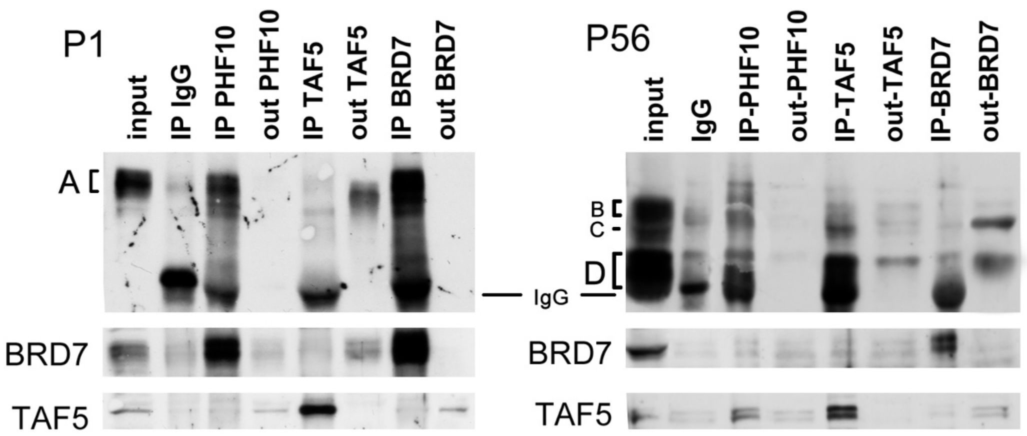

Figure S3. Western Blot of proteins precipitated with antibodies against PHF10 and BRD7 from P1 and P56 extracts. Western blot was developed with antibodies against PHF10 and BRD7 (indicated on the left). The obtained results show that antibodies against PHF10 co-precipitate BRD7 from P1 extract containing PHF10A isoform and PBAF complex. From P56 extract which contains PHF10D (associated with dcPBAF), the antibodies against PHF10 do not co-precipitate BRD7. These results are confirmed in reciprocal precipitation with antibodies against BRD7.

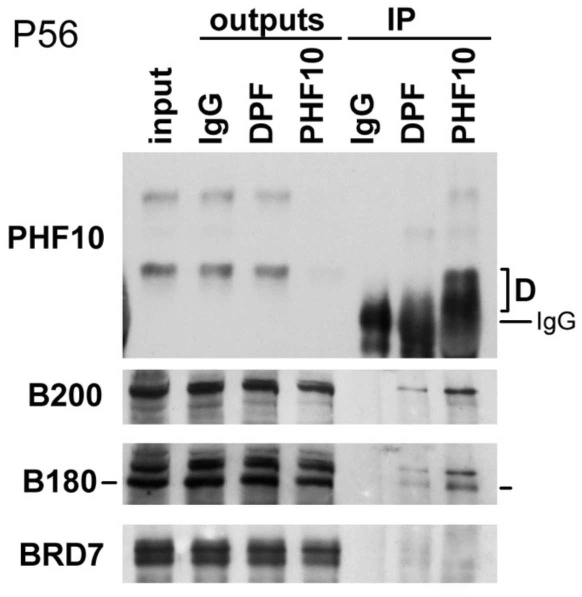

Figure S4. Western Blot analysis of the PBAF subunits precipitated with antibodies against DPF-domain and against total PHF10 from nuclear extracts of P56 murine brain. Anti-PHF10 antibodies precipitate PHF10D from P56 extract. They also co-precipitate BAF200, BAF180 but not BRD7, suggesting that BAF200 and BAF180 are associated with dcPBAF even in the absence of BRD7. Note that the Input on Supplementary Fig. 5 differs from Input on Supplementary Fig. 6 due to the different amount of extract loaded per lane.

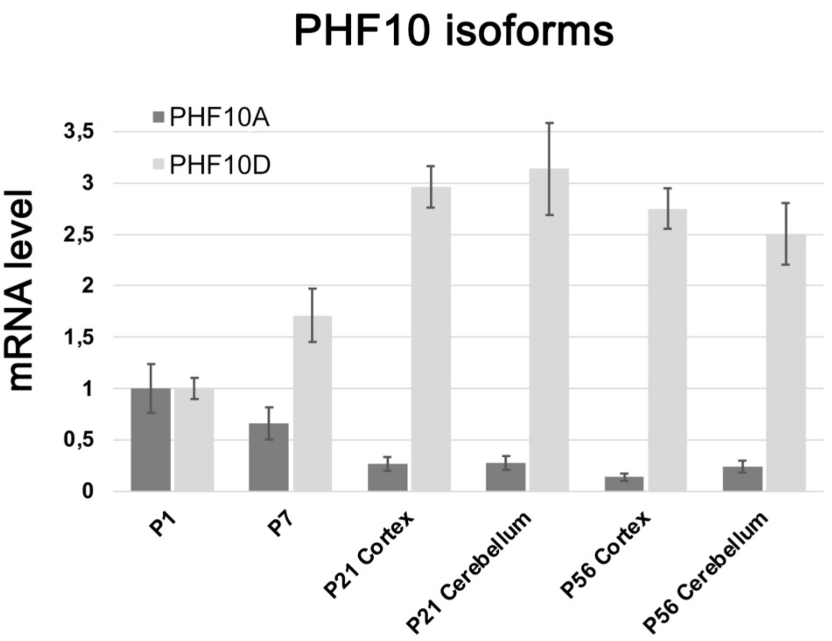

Figure S5. The levels of *PHF10A* and *PHF10A* isoforms in murine postnatal of brain. The levels of *PHF10A* and *PHF10D* isoforms during development were calculated relatively P1 expression. The values were normalized to the *actin* and *B2M* housekeeping genes according to MIQE guidelines (Bustin et al., 2009). At least three independent experiments were performed; values are mean ± SD.

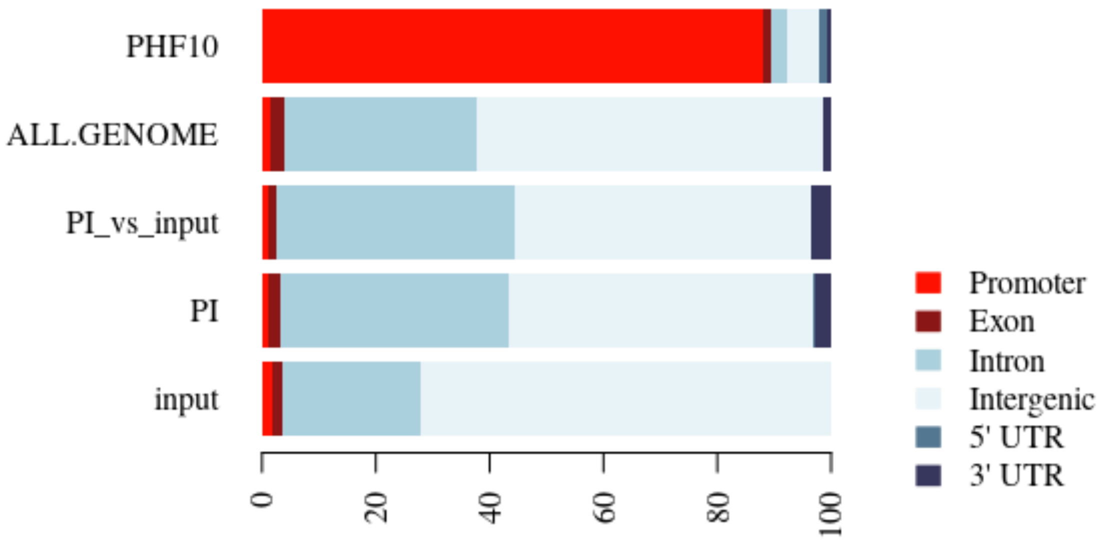

Figure S6. The distribution of genomic elements in the PHF10 binding sites and in the random sites distributed uniformly along the genome (N=301).

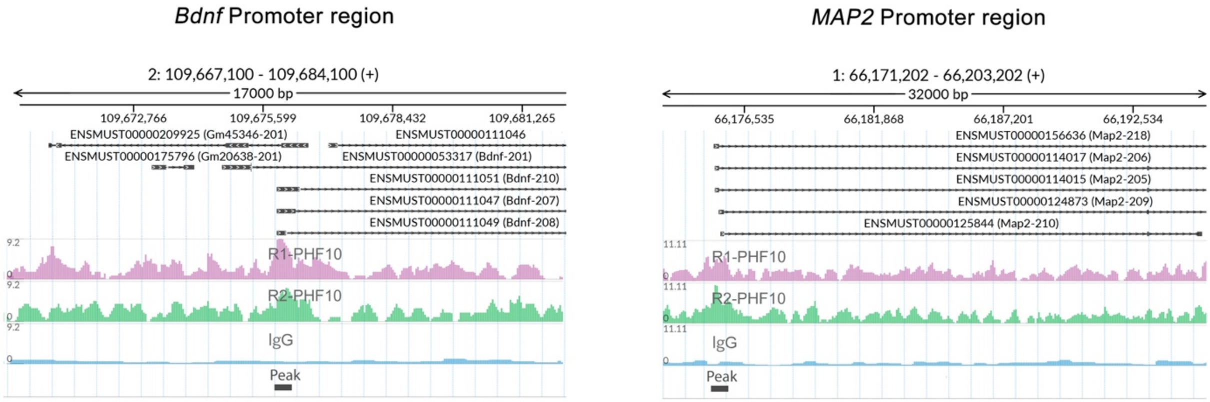

Figure S7. The results of ChIPseq showing the peaks of PHF10 from replicates and IgG (PI) at the promoters of mouse neuronal genes *Bdnf* and *MAP2* homologues to human genes shown at Figure 6.

**Housekeeping genes**		**Neuron-specific genes**	
Calm1	Kdm5a	Ckb	Tprn
Tcf25	Nsmce1	Ikzf4	Tenm2
Ykt6	Fus	Elavl3	Map1b
Calr	D230025D16Rik	Clstn3	Scrt2
Ulk2	Spg11	Eef1a2	Ccdc62
Jkamp	Cdk10	Cntnap1	Rpl7a
Kctd20	Chmp2a	Rmnd1	Kcnc3
Ctnnb1	Exoc3	Vegfa	Tuba1a
Zfp655	Map2k2	Nrxn1	Hist4h4
H3f3b	Zdhhc17	Celf4	
Zfp207	Nufip2	Cabyr	
Ywhah	Tmem183a	Rbfox3	
Rab21	Mgat2	Nptx1	
Yeats4	Saysd1	Tmeff2	
Hint1	B230219D22Rik	Efcab2	
Hnrnpab	Tmco1	Stmn1	
Canx	Mat2a	Errfi1	
Dbn1	Cd47	Nsg1	
Cox11	Tsnax	Acad10	
Dnajc27	Rpl32	Uncx	
Ddx5	Zc3h4	Slc25a4	
Pafah1b1	Taok2	Zic1	
Ube2g1	BC003965	Shf	
Tmx1	Chrac1	Neurod1	
Btf3	D2hgdh	Arpp21	
Sub1	Arih1	Lrrn1	
Eif4a2	Rabgef1	Dusp5	
Denr	Hspa5	Ints6	
Hsp90ab1	Gpvd1	Zic4	

Brd4	Psmd5	Cpeb2
Cmtr1	Pdia3	Atp1b2
Srsf7	Nop56	Nrep
Ring1	Arfgap1	Cnr1
Ercc3	Raly	Saysd1
Chuk	Slc35a1	Insig1
Ssbp1	Trnau1ap	Gap43
Gfpt1	Rab35	Gpr85

Table S3. Housekeeping and neuron-specific genes controlled by dcPBAF in adult (120P) mouse brain.

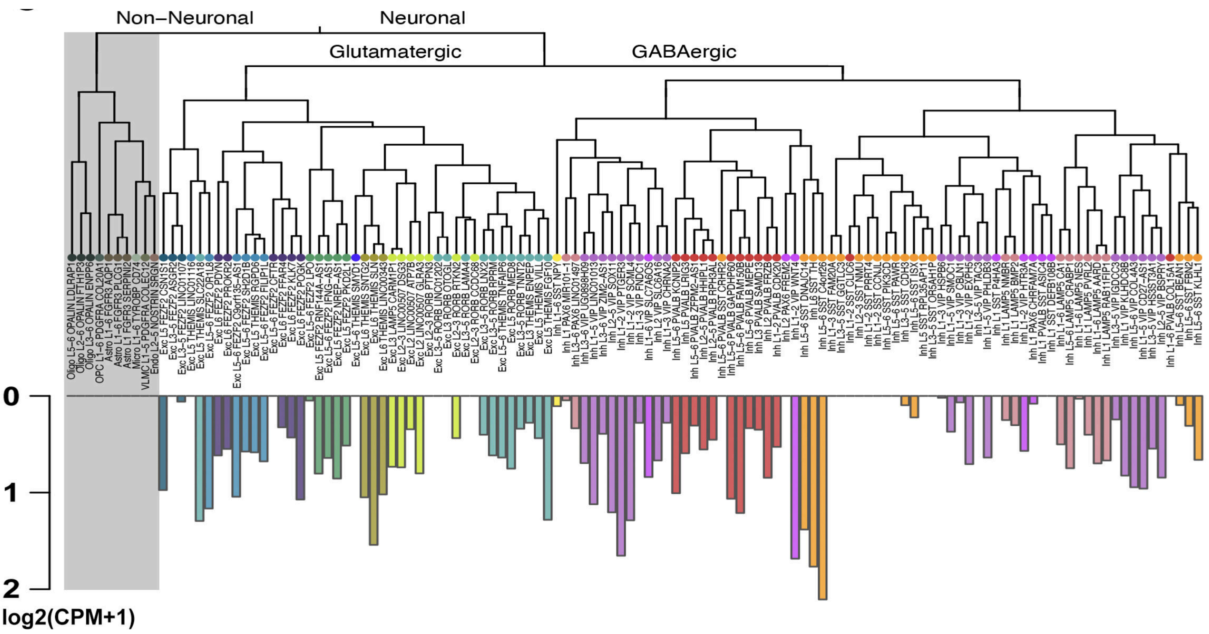

Figure S8 Trimmed mean expression (25%-75%, log2(CPM+1)) of PHF10 in different cell types, hierarchically clustered based on the gene expression data provided by the Allen Brain Atlas (https://portal.brain-map.org/atlases-and-data/rnaseq/human-m1-10x) (Sunkin et al. 2013; Bakken et al. 2021).

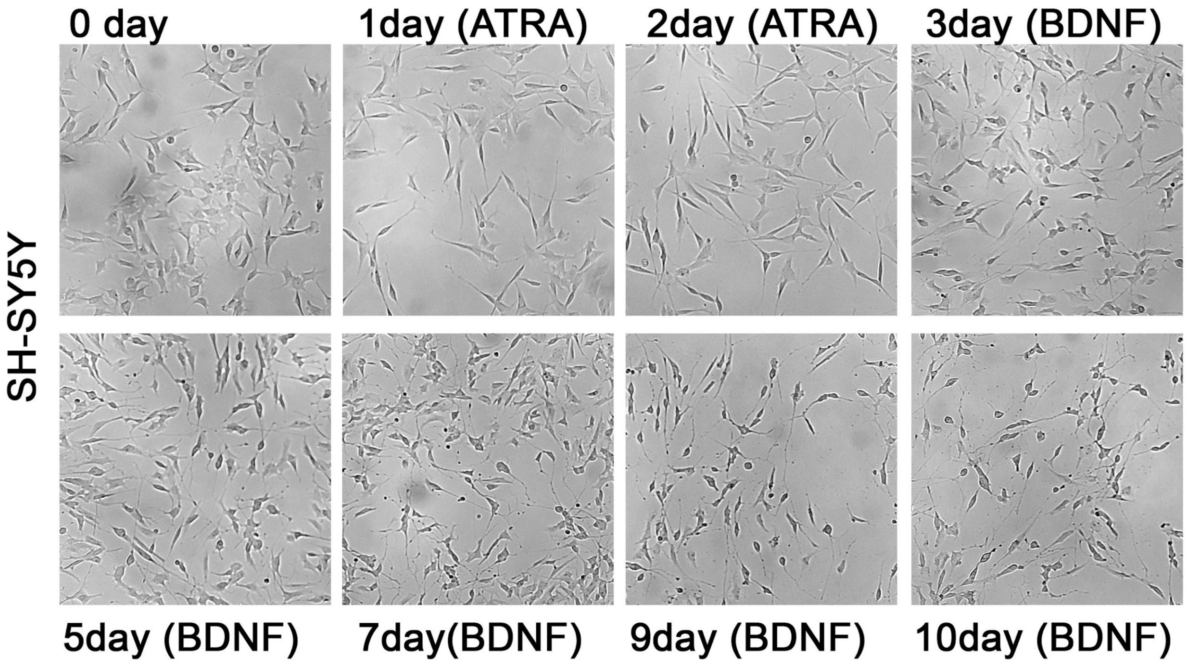

Figure S9. SH-SY5Y differentiation after ATRA and BDNF treatment during 10 days

Table S4. siRNA sequences for PHF10D and Control knockdowns in SH-SY5Y cells.

**Name of siRNA**	**Sequence (5'-3')**
PHF10-D_siRNAi(I)_for	GCUGUACAGUUUCAUAUUdTdT
PHF10-D_siRNAi(I)_rev	AAUAUGAAACUGUACAGCdTdT
PHF10-D_siRNA(II)_for	GCUAAAGGUCAUAGUUUAdTdT
PHF10-D_siRNA(II)_rev	UAAACUAUGACCUUUAGCdTdT
Control_siRNA_for	AGGUCGAACUACGGGUCAAdTdC
Control_siRNA_rev	UUGACCCGUAGUUCGACCUdAdG

Table S5. Sequences of primers for mRNA measurement.

**Name of mRNA Primers**	**Sequence (5'-3')**
RPLP0_mRNA_for	ACTGGAGACAAAGTGGGAGCC
RPLP0_mRNA_rev	CAGACACTGGCAACATTGCG

PHF10-total_mRNA_for	CCGGGAACGCATGGAAGAAAG
PHF10-total_mRNA_rev	CACCATCACTGTCTAGAGCAGGGAGC
PHF10-A_mRNA_for	CCAGGGAAGACAGAAATCAAAAGAC
PHF10-A_mRNA_rev	CCATTGTCATATCCAGGCAAGAAGG
PHF10-D_mRNA_for	CCAGGGAAGACAGAAATCAAAAGAC
PHF10-D_mRNA_rev	CAGGGGCTTTTTTCTTCTACCTTG
BDNF_mRNA_for	GGAGCTGAGCGTGTGTGACAG
BDNF_mRNA_rev	GGGATTGCACTTGGTCTCGTAG
MAP2_mRNA_for	GCTCATCATGTACCTGGAGGTG
MAP2_mRNA_rev	GGTGATGCCACGCTGGATCTG
TrkB_mRNA_for	CTGTAGTGTGGCAGGTGATCCG
TrkB_mRNA_rev	GATCTGCTTCCCACTGTCATCG
NREP_mRNA_for	TTGTCTGGGTCAGTCAAGAACC
NREP_mRNA_rev	GAGCGGAGTTCACTGCTGC
ELAVL3_mRNA_for	ACCATCAGACCCAGCGTTTCC
ELAVL3_mRNA_rev	GCACGCTCTCGTCTGCCTCCG
EEF1A2_mRNA_for	CGTGTCGGTGAAGGACATCCG
EEF1A2_mRNA_rev	CAGCTCCGCAAACTTGCAGG
PHF10-total_mRNA(Mm)_for	CGGGAGCGCATGGAAGAAAG
PHF10-total_mRNA(Mm)_rev	CCATCACTATCTAGTGCCGGGAGC
PHF10-A_mRNA(Mm)_for	GGACACCTTCCACGGAAGACAG
PHF10-A_mRNA(Mm)_rev	CATATCCAAGCAAGAAGGGTGGC
PHF10-D_mRNA(Mm)_for	GGACACCTTCCACGGAAGACAG
PHF10-D_mRNA(Mm)_rev	CAGGGGCTTTTTTCTTCTACCTTG
B2M_mRNA(Mm)_for	CGGGAGCAGGTGGACCAGGG
B2M_mRNA(Mm)_rev	GTGTCCAGTAGTCGTGTGATGAGGTG

actin_mRNA(Mm)_for	GGCACCACACCTTCTACAATGAGC
actin_mRNA(Mm)_rev	CCAGAGGCATACAGGGACAGC

Table S5. Sequences of primers for ChIP.

**Name of ChIP Primers**	**Sequence (5'-3')**
BDNF_ChIP_for	GCCGTTTGACCAATCGAAGC
BDNF_ChIP_rev	GCGGGACAGCGAGCGGGC
MAP2-1_ChIP_for	GCCGAGGCGGAGCTGCTGCG
MAP2-1_ChIP_rev	GAAGCGGAAGGGAAGTAAAGG
MAP2-2_ChIP_for	ACTGTTTCTCTATTTAAGCGGTG
MAP2-2_ChIP_rev	CAGGAGTCGAGTCTATCAGCC
TrkB_ChIP_for	CTCCCTGGTGCTTTTGTCTGG
TrkB_ChIP_rev	GCGGGACAGCGAGCGGGC
NREP_ChIP_for	GGTGTATATTCCTGACTCCCC
NREP_ChIP_rev	ACCGTGTAGTGTAATGCCTCAG
ELAVL3_ChIP_for	GATTCAGAGTCCCGACCAGTG
ELAVL3_ChIP_rev	CGGTCCCGTGTGTTCAAGTC
EEF1A2_ChIP_for	GATGTCGTGTACTGGCTCCGC
EEF1A2_ChIP_rev	CGGCACTTACCTGTGTTCTGG
Control_ChIP_for	GCTCCTGAAGTCCTGCCTCTTG
Control_ChIP_rev	GGTGCTTTGCAGCAGATGCC

Methods

Immunoprecipitation

For immunoprecipitation, cerebellum or whole brain were homogenized on ice in PBS buffer supplemented with PIC in Dounce Loose (Millipore), centrifuged at 1000*g*, 4˚C for 10 minutes.

Then the pellet was washed 3 times by 10V of PBS each, also supplemented with PIC, resuspend in Lysis Buffer (50 mM HEPES-KOH pH 7.9; 140 mM NaCl; 1 mM EDTA; 1% Triton X-100; 0.1% Na deoxycholate; 0.1% SDS; PIC and PhIC) and homogenized in Dounce Tight on ice. After sonication, the debris was centrifuged at 4˚C, 13.2 krpm for 15 minutes. Supernatant was mixed with antibodies (10 µl antibodies against PHF10 and TAF5 per IP) and Protein A Sepharose (Sigma). Beads were washed twice with IP-500 buffer (25 mM Tris-HCl pH 7.9; 5 mM $MgCl_2$; 10% glycerol; 500 mM NaCl; 0.1% NP-40; PIC; PhIC) and once with IP-100 buffer (25 mM Tris-HCl pH 7.9; 5 mM $MgCl_2$; 10% glycerol; 100 mM NaCl; 0.1% NP-40; PIC; PhIC). Precipitated proteins were eluted with 4× Laemmli buffer (200 mM Tris-HCl pH 6.8; 4% SDS; 40% glycerol; 0.01% bromophenol blue; 100 mM DTT) and boiled for 10 min.
